# Association of Growth Hormone Receptor Gene Polymorphisms (rs6180, rs6182, rs6184) with Skeletal Class III Malocclusion and Prognathic Mandibles in the Deutero-Malay Race

**DOI:** 10.1055/s-0044-1801279

**Published:** 2025-05-01

**Authors:** Seto Adiantoro Sadputranto, Ani Melani Maskoen, Avi Laviana, Endang Sjamsudin, Arlette S. Setiawan

**Affiliations:** 1 Department of Oral Maxillofacial Surgery Hasan Sadikin General Hospital, Faculty of Dentistry, Padjadjaran University, Bandung, Indonesia; 2 Department of Oral Biology, Faculty of Dentistry, Padjadjaran University, Bandung, Indonesia; 3 Department of Orthodontic, Faculty of Dentistry, Padjadjaran University, Bandung, Indonesia; 4 Department of Oral Maxillofacial Surgeon, Faculty of Dentistry, Padjadjaran University, Bandung, Indonesia; 5 Department of Paediatric Dentistry, Universitas Padjadjaran, Bandung, Indonesia

**Keywords:** prognathic mandible, growth hormone receptor, single-nucleotide polymorphism, Deutero-Malay race

## Abstract

**Objective** A prognathic mandible, characterized by the anterior protrusion of the mandible, is often associated with skeletal class III malocclusion. Polymorphisms rs6180, rs6182, and rs6184 in the growth hormone receptor (GHR) gene are thought to influence the development of this condition among various races. This study explores the link between these GHR gene polymorphisms and class III skeletal malocclusion in individuals with prognathic mandibles within the Deutero-Malay race.

**Materials and Methods** Saliva samples from 104 participants were analyzed using polymerase chain reaction sequencing. The association between GHR polymorphisms and malocclusion phenotype was examined using the chi-square statistical method.

**Results** Significant differences were observed in the rs6180 AC and CC genotypes between control and outcome groups, with a *p* -value of 0.023 and an odds ratio of 1.667, suggesting a notable association.

**Discussion** The rs6180 polymorphism in the GHR gene appears to alter protein structure and function, promoting mandibular growth in the anteroposterior direction among the Deutero-Malay race.

**Conclusion** The rs6180 polymorphism in the GHR gene is significantly associated with the occurrence of skeletal class III malocclusion accompanied by prognathic mandibles in the Deutero-Malay race.

## Introduction

Mandibular prognathism, often associated with skeletal class III malocclusion, arises from a complex interplay of genetic and environmental factors. The significant genetic component in its etiology is underscored by familial studies, such as those in the Habsburg family, where a high incidence of mandibular prognathism was documented. Liu et al, in 2017, emphasized that both hereditary traits and environmental influences contribute to these conditions. Environmental factors may include trauma, habitual mandibular advancement, pituitary disorders, congenital anatomical anomalies, enlarged tonsils, nasal breathing difficulties, and hormonal imbalances. 2 3 4 5

Growth hormone (GH), a protein produced by somatotropic cells in the anterior pituitary under hypothalamic control, plays a crucial role in metabolism and bone growth. GH stimulates osteoblasts' proliferation, differentiation, and activity, leading to the expression of bone morphogenetic proteins 2 and 4. These proteins facilitate the maturation of osteoprogenitor cells. GH binds to the GH receptor (GHR) on target tissues, initiating a signaling cascade essential for bone development. Any polymorphism in the GHR can alter the interaction with GH, potentially affecting its functional outcomes. 6 7

Single-nucleotide polymorphism, or SNP, is a variation of a single nucleotide base sequence deoxyribonucleic acid (DNA) (C/T or g/A) or nucleotide base pair (C/g, A/T, C/A, or T/G) in one different genome in each individual and inherited. SNP was found to be involved in prognathism mandibular, based on the Human Gene Mutation Database, there were 56 GHR gene mutations. In several studies on the effects of the GHR gene mutation in the craniofacial region, including research on the ethnic Chinese Han, there are polymorphisms in the codon 526 region, causing changes in the length of the mandible ramus measured from the condyle to the gonion or from the lower jaw to the gonion (co-go/ar-go). The position of nucleotide to 1777 in the GHR gene, there is a transversion from cytosine to adenine, converting amino acids in codon 561 from proline to trise (p561t), which has an impact on the cytoplasmic domain of the GHR gene. The Japanese population does not have a significant correlation between height and the P561T GHR gene variant, but there is a long relationship between mandible ramus. The P561T variant carrier will significantly have a shorter mandibular ramus (Co-GO) length than the shape of the wild-type in the locus. This discovery states that the cytoplasmic change of the GHR gene produced by P561T has no significant role in the final result of a body height and only has an effect on the growth of the mandibular, but the effect of P561T on craniofacial growth in children is not yet known with certainty. 8 9 10 11

Another study in the Japanese, Korean, Turkish, Iranian, and Chinese populations found that the SNP RS6180, RS6182, and RS6184 GHR gene were related to the height of mandibular ramus, but in the Deutero-Malay population, there was no reporting. 11 12

The Indonesian population is composed of various kinds of races that have been passed down for generations forming several of ethnic groups, which are divided into three groups, namely, the Malay-Proto subrace and the Deutero-Malay subrace, which is classified in the Malayan Mongoloid subrace, while the Chinese tribes are classified in the Asiatic Mongoloid subrace. Most of the Indonesian population is dominated by the Paleo-Mongoloid race, better known as the Malay race, which is then divided into Malay-Deutero and Malay-Proto. The Deutero-Malay population consists of Aceh, Minangkabau, Bugis, Makassar, Sundanese, Javanese, Bali, Malay, and Betawi. The Deutero-Malay population's characteristics include brown skin, straight hair, slender tall body, the shape of the mouth and nose is medium, and the jawbone is smaller than the Malay-Proto populatyion. 13 14

The interaction of GH with its receptor (GHR) on target cells triggers an intracellular signaling pathway essential for bone growth. Variations or polymorphisms in the GHR can modify this interaction, potentially impacting the function and effectiveness of GH. 8 9 11 Since the Deutero-Malay race is the most numerous in Indonesia, this study will concentrate on studying these SNPs in the Deutero-Malay population to uncover the etiopathogenesis of skeletal class III malocclusion with mandibular prognosis.

## Materials and Methods

This study focused on the Deutero-Malay population exhibiting class III skeletal malocclusion with prognathic mandibles in private practices and specialized orthodontic and oral surgery clinics and departments in Bandung, Semarang, and Jakarta. Participants were recruited through consecutive sampling. This research has received approval from the Universitas Padjadjaran Health Research Ethics Committee with document number 38/UN6.KEP/EC/2021.

The eligible participants were categorized into two groups: the case group and the control group. The criteria for participants in the case group included individuals aged 19 to 40 with skeletal class III malocclusion and prognathic mandibles, as confirmed by cephalometric examination. Participants must not have congenital or systemic diseases that could influence growth and development, such as cleft lip and palate, Down syndrome, or GH disorders. They must have never experienced facial or mandibular trauma. The control group consisted of people with a normal class I skeletal connection, while those with class II malocclusion were omitted. Participants chosen from both groups were educated and provided informed consent.

### Materials

This study primarily utilized saliva obtained from participants from the control and case groups. DNA samples were obtained from the buccal mucosa utilizing the Buccal Swab DNA Extraction Kit. The samples were duplicated using the polymerase chain reaction (PCR) method, an enzymatic technique for DNA amplification. The analysis of DNA comprised multiple stages:

1. Saliva collection to analyze polymorphisms rs6180, rs6182, and rs6184.
2. Isolation of DNA from acquired samples.

DNA isolation was conducted to acquire a DNA pellet from a saliva sample collected. The gathered sample was centrifuged to acquire a cell pellet. The harvested cell was subsequently treated with several treatments to yield the DNA pellet ultimately.

3. Implementation of PCR protocols.

The collected DNA pellet was subsequently amplified using the PCR protocol to obtain a larger sample.

4. DNA sequencing via the Sanger method.

Sanger sequencing incorporated dideoxynucleoside (ddNTP) alongside normal nucleotide (NTP).

The primary that was used for rs6180, rs6182, and rs6184 is:
Forward: 5′ CGTACCAGCTGTTGTGAACC 3′
Reverse: 3′ AGCAAAGAATTGACTGGGGC 5′
Location of rs6180, rs6182, and rs6184 was based on acquired data of Asia.ensemble.

Additionally, cephalometric radiographs were used to assess each participant's craniofacial structure. The OneCeph digital cephalometric tracer and an Android smartphone application made tracing and measurements easier. This application was specifically developed to calculate the values of Sella-Nasion-A point (SNA), Sella-Nasion-B point (SNB), and A point-nasion-B point (ANB) angles, which are important in assessing craniofacial relationships.

### Statistical Analysis

The chi-square test was used to compare the differences between the GHR gene polymorphism groups in both groups, using a nominal scale. The data are processed SPSS V.29 (IBM).

## Result

The study was conducted between June 2021 and March 2022, involving 104 participants who satisfied the inclusion criteria. The participants were allocated into a case group of 47 and a control group of 57. All subjects were patients from multiple medical facilities, including the maxillofacial oral surgery and orthodontic specialist departments at Hasan Sadikin Hospital, Universitas Padjadjaran Dental Hospital, and private clinics in Bandung and Semarang. Initial assessments utilized the OneCeph software to compute cephalometric values, specifically SNA, SNB, and ANB. Among the total subjects, there were 53 men, comprising 27 (26%) in the case group and 26 (25%) in the control group. Of the remaining 51 subjects, 20 (19.2%) were women in the case group while 31 (29.8%) were women in the control group. Statistical analysis employing the chi-square test yielded a probability value of 0.230, suggesting no significant association between gender and class III skeletal malocclusion with mandibular prognathism ( *p* = 0.230 > 0.05). The findings are presented in Table 1 .

**Table 1:** Data on research subject characteristics

Variable	Group				Total		*p* -Value
	Case		Control				
Gender	***N***	**%**	***N***	**%**	***N***	**%**	0.230
Male Female Subtotal	27 20 47	26.0 19.2	26 31 57	25.0 29.8	53 51 104	51.0 49.0 100.0	
Age	***N***	**%**	***N***	**%**			0.167
17–25 y 26–35 y 36–45 y Subtotal	26 20 1 47	25.0 19.2 1.0 45.2	23 29 5 57	22.1 28.9 4.8 54.8	49 49 6 104		

**Fig. 1:**
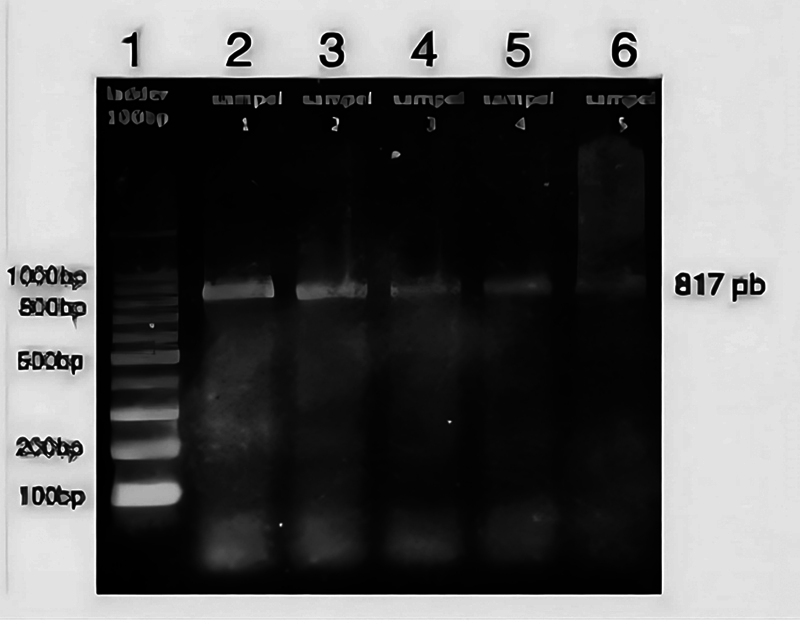
Electrophoresis image of polymerase chain reaction (PCR) product of 817 base pairs (bp), lane 1. Molecular weight marker, ladder 100, lanes 2–6.

This study adhered to age criteria established by the Indonesian Ministry of Health in 2009, which categorizes age groups as follows: toddlerhood (0–5 years), childhood (6–11 years), early adolescence (12–16 years), late adolescence (17–25 years), early adulthood (26–35 years), mid-adulthood (36–45 years), late adulthood (46–55 years), and old age (56 years and above). PCR sequencing was employed to analyze the GHR gene polymorphisms rs6180, rs6182, and rs6184 in 118 samples. The results were visualized using electrophoresis, as shown in Fig. 1 .

**Fig. 2:**
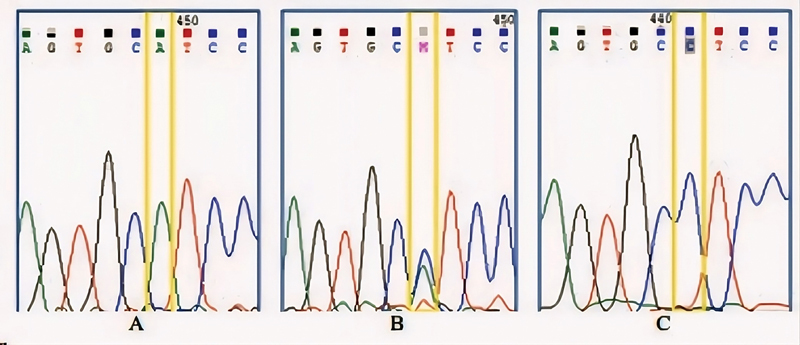
Electropherogram image of rs6180 growth hormone receptor (GHR) gene. ( **A** ) AA genotype, ( **B** ) AC genotype, and ( **C** ) CC genotype.

The PCR products for the rs6180 polymorphism include two alleles: allele A (normal) and allele C (mutant/polymorphism). There are three possible genotypes for rs6180: AA, AC, and CC. Fig. 2 displays an electropherogram image from the sequencing of the rs6180 region. This visualization aids in identifying the genotypes based on color-coded graphs: the AA genotype is represented by a single green graph, the AC genotype by a combined green and blue graph, and the CC genotype by a single blue graph ( Table 2 ).

**Table 2:** The frequency of allele and genotypes of the GHR RS6180 gene

Allele/genotype	Control ( *n* )	Case ( *n* )	Total ( *n* )	Chi-square	*p* -Value	OR
GHR rs6180				2.564	0.109	1.567
Allele						
A (normal) C (mutant) Total	60 54 114	39 55 94	99 109 208			
Genotype				7.520	0.023	1.667
AA (normal) AC (heterozygote) CC (mutant) Total	21 18 18 57	7 25 15 47	28 43 33 104			
Genotype				6.307	0.012	3.333
AA AC + CC Total	21 36 57	7 40 47	28 76 104			

Abbreviations: GHR, growth hormone receptor; OR, odds ratio.

### rs6180 (A- > C)

rs6180 consists of alleles A (normal) and C (mutant/polymorphism), with three possible genotypes: AA, AC, and CC. There are 60 instances of allele A in the control group, compared with 39 in the case group. Conversely, the mutant allele C (A- > C) appears 55 times in the case group and 54 times in the control group. The *p* -value for allele RS6180 is approximately 5.0 × 10^–109, indicating no significant difference in the distribution of allele RS6180 between the case and control groups.

Regarding genotype distribution, the total number of mutant genotypes (AC and CC) combined is 76, while the normal genotype (AA) counts for 28 instances. The *p* -value for the mutant genotype is 0.012, which is less than 0.05, and the chi-squared value is 6.307. These results suggest a significant difference between the control and case groups in the distribution of GHR RS6180 genotypes. The odds ratio for the risk associated with mutant genotypes contributing to class III skeletal malocclusion with mandibular prognathism is approximately 3.333.

### rs6182 (G- > T)

**Fig. 3:**
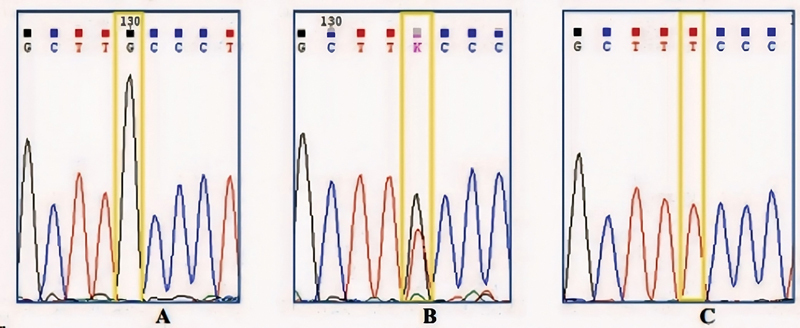
Overview of electropherogram image of rs6182 growth hormone receptor (GHR) gene. ( **A** ) GG genotype, ( **B** ) genotype GT, and ( **C** ) genotype TT.

The genetic structure of allele plate number 1 comprises two types of alleles: G (normal) and T (mutant polymorphism). Three possible genotypes are derived from these alleles: GG, GT, and TT. Fig. 3 presents an electropherogram of the sequence region from plate number 1. This visualization helps identify the genotypes: the GG genotype is shown as a single black-colored graph, a combined black and red-colored graph depicts the GT genotype, and a single red-colored graph represents the TT genotype. Fig. 3 displays two graphs, black (G) and red (T), illustrating the polymorphism or genetic variation from G to T, thus indicating the presence of the GT genotype, a heterozygous mutant. Fig. 3C presents a single red graph (T), signifying a polymorphism or genetic variation from G to T, resulting in the TT genotype, which is a homozygous mutant.

**Fig. 4:**
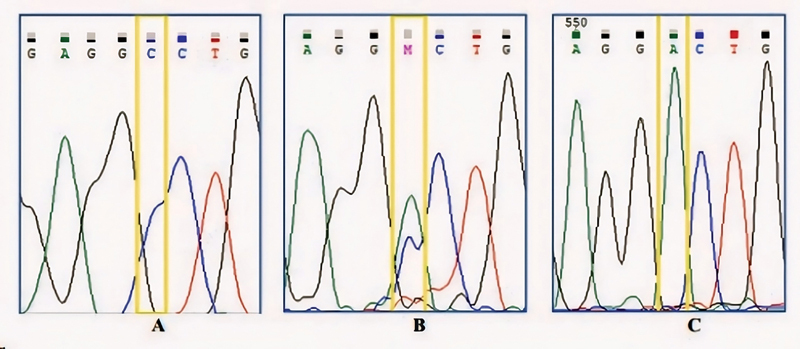
Electropherogram image of rs6184 growth hormone receptor (GHR) gene. ( **A** ) CC genotype, ( **B** ) genotype CA, and ( **C** ) genotype AA.

For allele RS6182, the distribution in the study groups is as follows: the normal allele G appears 91 times in the control group and 83 times in the case group. The mutant allele T is found 23 times in the control group and 11 times in the case group. With a *p* -value of 0.100, there is no statistically significant difference between the case and control groups, as the value exceeds the threshold of 0.05 ( Table 3 ). For RS6184, the alleles consist of C (normal) and A (mutant/polymorphism), with possible genotypes of CC, CA, and AA. Fig. 4 presents the electropherogram results for the RS6184 region, showing the CC genotype as a single blue graph, the CA genotype as a combined blue and green graph, and the AA genotype as a single green graph.

**Table 3:** The frequency of allele and genotypes of GHR rs6182 gene

Allele/genotype	Control ( *n* )	Case ( *n* )	Total ( *n* )	Chi-square	*p* -Value	OR
GHR rs6182				2.705	0.100	0.524
Allele						
A (normal) C (mutant) Total	91 23 114	83 11 94	174 34 208			
Genotype				0.029	0.986	1.067
AA (normal) AC (heterozygote) CC (mutant) Total	38 15 4 57	32 12 3 47	70 27 7 104			
Genotype				1.237	0.266	0.611
AA AC + CC Total	38 19 57	36 11 47	74 30 104			

Abbreviations: GHR, growth hormone receptor; OR, odds ratio.

In the allele RS6184 analysis, the normal allele C is present 89 times in the control group and 76 times in the case group, totaling 165 instances. The mutant allele A appears 25 times in the control group and 18 times in the case group, with a combined total of 43 instances. The *p* -value calculated for this allele distribution is 0.622, indicating no statistically significant difference between the control and case groups, as the value exceeds 0.05. These findings are summarized in Table 4 .

**Table 4:** Frequency of allele and genotype GHR gene RS6184

Allele/genotype	Control ( *n* )	Case ( *n* )	Total ( *n* )	Chi-square	*p* -Value	OR
GHR rs6184				0.243	0.622	0.843
Allele						
A (normal) C (mutant) Total	89 25 114	76 18 94	165 43 208			
Genotype				0.980	0.741	1.846
AA (normal) AC (heterozygote) CC (mutant) Total	38 13 6 57	32 12 3 47	70 25 9 88			
Genotype				0.024	0.878	0.938
AA AC + CC Total	38 19 57	32 15 47	70 34 88			

Abbreviations: GHR, growth hormone receptor; OR, odds ratio.

Statistical analysis was performed using the chi-square test, with the following hypothesis: H0 = no difference in the proportion of genotypes/alleles between the case and control groups. The analysis revealed varying results for different polymorphisms:

- Allele rs6180: The *p* -value was 0.109, more significant than the significance level of 0.05, suggesting no statistical difference in allele frequencies between the case and control groups.
- Genotype rs6180: The *p* -value was 0.023, and the chi-square value was 7.520, indicating a significant difference between the groups. The odds ratio of 1.667 suggests that individuals with the mutant genotype are 1.667 times more likely to exhibit the case condition, with a confidence interval ranging from 0.668 to 4.160.
- Genotype mutant rs6180: With a *p* -value of 0.012, this genotype shows a significant difference between the groups. The odds ratio of 3.333 indicates that individuals with this genotype are 3.333 times more likely to be affected, with a risk range of 1.268 to 8.764.

This implies that the GHR rs6180 polymorphism significantly increases the risk of class III skeletal malocclusion with prognathic mandibles in the Deutero-Malay race. However, the alleles rs6182 and rs6184, with *p* -values greater than 0.05, do not show significant differences between the case and control groups, suggesting they are not risk factors for this condition.

Furthermore, the study also noted measurements related to the mandibular structure:

- *Mandibular ramus length (ar-go)* is labeled as measurement number 2.
- *Mandibular corpus length (co-po)* is labeled as measurement number 3.
- *Mandibular angle (gonion)* : This is calculated at 25 degrees, determined by the intersection of the tangent lines along the lower edge and the back of the mandible, known as the gonion angle.

## Discussion

Class III skeletal malocclusion with mandibular prognathism describes a specific dental and skeletal relationship where the mandible is positioned anteriorly relative to the maxilla and cranial base, often resulting in a protruding jawline. This condition is associated with various complications, including temporomandibular joint disorders, particularly in cases of facial asymmetry. Research by Hillary Lathrop-Marhal et al in 2021 highlighted that dentofacial disharmony could elevate the risk of auditory distortions, affect visual perception, and cause pronunciation difficulties with certain consonants depending on the extent of the misalignment. 15 Additionally, abnormalities in the growth of the mandibular condyle can lead to changes in the jaw structure, further exacerbating facial asymmetry. 11 A 2017 study by Lee et al utilizing three-dimensional imaging techniques revealed that individuals with prognathic mandibles and facial asymmetry often exhibit an imbalanced facial contour, particularly in the posterior-anterior direction. 16

GH binds to the GHR when it enters the bloodstream, located on the surface of cells in condyle cartilage. This interaction activates the Janus kinase and signal transducer and activator of transcription pathways. Activating these pathways leads to the replication of transcription and DNA translation within the cell nucleus, which is essential for cellular growth and development. 17 18

GHR belongs to the type 1 cytokine receptor family and includes an extracellular domain (ECD). It plays a crucial role in craniofacial skeletal development by triggering a series of growth processes through these signaling pathways. Interestingly, GHR affects the mandibular bone, an essential component of the craniofacial skeleton in endochondral development sites like the mandible. 17 18

GHR is distributed on cell surfaces throughout the body and encoded by a gene located at 5P13. The gene structure includes 10 exons, with 9 being coding exons. Exon 2 encodes the signal peptide, exons 3 to 7 comprise the ECDs, exon 8 is responsible for the transmembrane region, and exons 9 and 10 comprise the intracellular domains. 17 18

Several studies have established a link between genetic variations and prognathic mandibles. Notably, research has identified a connection between the length of the mandibular ramus and GHR polymorphisms. For instance, studies involving the Han tribe in China found that GHR polymorphisms correlate with the mandible ramus dimensions. Similar findings have been reported in Korea, where relationships between polymorphisms and the length of the mandibular ramus have been observed across various studies. 17 18

Specifically, GHR polymorphisms such as i526L (plate number 2, document number 2), along with other documented variations (document numbers 1 and 3), have been associated with class III skeletal malocclusion characterized by significant ramus elongation and corpus mandibula extension. The GHR gene plays a crucial role in the growth of the mandibular Matn1 gene and also influences the synthesis of Matrilin-1, which is essential for endochondral frame development. The mandibular condyle's endochondral surface binds to GH, activating GH and promoting mandibular growth. Thus, GHR polymorphism can enhance GH sensitivity, leading to class III skeletal malocclusion with a prognathic mandible. 17 18

Further research, such as a study by Jang et al 19 on the Korean population, illustrates that MATN1 polymorphisms (specifically 7987 G > A and 8572 C > T) significantly contribute to mandibular development. These polymorphisms in the MATN1 gene represent a risk factor for developing class III skeletal malocclusion with a prognathic mandible. Additionally, genetic variations, including a T to C transition in exon 5 found in the Deuteron-Malay race, along with polymorphisms in MYOI, MATN1, and RUNX2, are associated with the occurrence of class III skeletal malocclusion with a prognathic mandible. 17 18 20

The study results indicate that the SNP rs6180 influences mandibular growth predominantly in the anteroposterior direction rather than contributing to a more extended facial profile. The facial plane typically helps illustrate variations in facial profiles to determine the growth direction and development patterns. The mandibular plane angle (MP-SN), formed between the mandibular and cranial baselines, typically ranges from 230 to 370 degrees. The growth behaviors of the maxilla and mandible influence development patterns in this vertical direction. Head cephalogram examinations identified three distinct facial profile groups based on this angle: (1) hypodivergent (SN-MP less than 270 degrees); (2) normodivergent (SN-MP between 230 and 370 degrees); and (3) hyperdivergent (SN-MP greater than 370 degrees).

The class III mandibular prognathism cases with SNP rs6180 AC and CC genotypes suggest that the majority of the case group with SNP RS6180 AC and CC genotypes exhibit mandibular growth more in the sagittal direction rather than vertically. The occlusal plane angle (OP-SN) between the cranial base (S.) and the occlusal plane (OP) values is typically around 140 degrees. Values greater than 140 degrees indicate excessive vertical growth, whereas values less than 140 indicate predominant horizontal growth. This suggests that the class III skeletal group with prognathic mandibles is likelier to exhibit a short-faced rather than a long-faced profile. Additionally, skeletal class III phenotype varies by gender and race, with the highest prevalence observed in East Asian populations such as Korea, China, and Japan. A study by Bayram et al revealed that individuals with the CA genotype at P516T exhibit a shorter mandibular length (Co-G) and a more short-faced profile (AS-Me) compared with those with the CC genotype in the Turkish population. 21

Several studies have explored the influence of genetic polymorphisms on mandibular morphology across different populations. Tobón-Arroyave et al researched the Colombian population and found that the A allele from the GHR gene at a specific SNP (plate number 1) produced significant horizontal variations in mandibular morphology. This genetic variation may be a prognostic indicator for class III framework profiles. 22

In China, Zhou et al's study revealed that individuals with the CC genotype at the i526L polymorphism (plate number 2) exhibited a significantly longer mandibular length (from condyle to gonion) than those with AC or AA genotypes. 23 Haplotype analysis identified a notable correlation with the height of the mandible, indicating that the i526L GHR polymorphism (plate number 2) is associated with mandibular height in the Han Chinese population. Similar observations were made in Japan, where Nakawaki et al found that the GHR polymorphism influenced the distance between the left and right coronoids. However, a different GHR polymorphism was linked to a shorter mandibular ramus. 8

Tomoyasu et al and Kang et al from Japan and Korea, respectively, noted that polymorphisms (plate numbers 3 and 1) were associated with variations in the height of the mandibular ramus. 12 24 Contrarily, Adel et al reported that in the Egyptian population, the polymorphisms did not correlate with any variations in mandibular form. 25

rs6180 polymorphism results in a substitution of isoleucine with leucine. 26 Leucine is commonly found in anchovy fish. 27 In Indonesia, where most of the population belongs to the Deuteron-Malay race, a specific type of fish is an everyday dietary staple. This context is relevant to our study investigating the impact of the GHR polymorphisms rs6180, rs6182, and rs6184 on mandibular development. These polymorphisms have been identified as risk factors for mandibular ramus lengthening in populations in China, Japan, and Korea. 20 21 22 23 However, similar studies in the Egyptian population have shown no association between the rs6180 and rs6184 polymorphisms and mandibular shape. 25

Our study involved 47 samples from skeletal class III cases with prognathic mandibles within the Deuteron-Malay population. The findings indicated that only the rs6180 polymorphism was a significant risk factor for this condition. This polymorphism results in a conformational change in the GHR protein, affecting its ability to bind the GH ligand. Such a disruption in GHR signaling can lead to altered sensitivity or resistance to GH, promoting increased growth of the mandibular corpus in the anteroposterior direction.

Interestingly, the rs6182 and rs6184 polymorphisms did not correlate with the occurrence of class III skeletal malocclusion with mandibular prognathism in the Deuteron-Malay population, suggesting that these variants may only be characteristic genetic variations within this demographic group. Despite extensive research on the genetic factors influencing skeletal class III malocclusion with prognathic mandibles in other populations, such as those in China, Korea, Japan, Turkey, and Egypt, studies on the Deuteron-Malay race have been limited. The results of this study provide a new understanding of the genetic landscape characteristic of the Deuteron-Malay race in Indonesia.

## Conclusion

Research has identified the rs6180 GHR polymorphism as a significant genetic risk factor for skeletal class III malocclusion with prognathic mandibles in the Deutero-Malay race. This finding highlights the influence of specific genetic variations on craniofacial development. However, other polymorphisms and haplotypes associated with this receptor have not been linked to these abnormalities, suggesting a selective impact of specific genetic factors.

Given the complexities of craniofacial growth, further studies are essential to explore the environmental influences contributing to the development of class III skeletal abnormalities. Factors such as adequate nutrition and beneficial habits are crucial for further investigation to understand their role alongside genetic predispositions. Additionally, there is a need for comprehensive research to identify other gene polymorphisms that influence the growth and development of the jaw, particularly in conditions like skeletal class III malocclusion with prognathic mandibles.
